# Flot-2 Expression Correlates with EGFR Levels and Poor Prognosis in Surgically Resected Non-Small Cell Lung Cancer

**DOI:** 10.1371/journal.pone.0132190

**Published:** 2015-07-10

**Authors:** Qiuyuan Wen, Weiyuan Wang, Shuzhou Chu, Jiadi Luo, Lingjiao Chen, Guiyuan Xie, Lina Xu, Meirong Li, Songqing Fan

**Affiliations:** 1 Department of Pathology, the Second Xiangya Hospital, Central South University, Changsha, Hunan, China; 2 Department of Oncology, the Second Xiangya Hospital, Central South University, Changsha, Hunan, China; INRS, CANADA

## Abstract

We previously reported that expression of Flotillin 2 (Flot-2), a protein isolated from caveolae/lipid raft domains, increased significantly in nasopharyngeal carcinoma (NPC) compared with normal tissues. Signal transduction through epidermal growth factor receptors (EGFR) and Flot-2 play an important role in cancer development, but their precise role in lung cancer has not been investigated. In this study, we have investigated the correlation between the expression of Flot-2 and EGFR, which increase significantly in non-small cell lung cancer (NSCLC) patients (n=352) compared with non-cancer tissues. Additionally, patients with advanced stages of NSCLC had higher positive expression of Flot-2 and EGFR than patients with early stages. NSCLC patients with increased expression of Flot-2 and EGFR had significantly less overall survival rates than patients with less expression of Flot-2 and EGFR. Taken together, our data suggest that increased expression of Flot-2 and EGFR in NSCLC patients is inversely proportional to the disease prognosis and that increased expression of Flot-2 associated with increased EGFR may serve as a biomarker to predict poor disease prognosis.

## Introduction

Lung cancer is one of the major causes of cancer-related mortality worldwide. There are about 80%-85% of lung cancer cases diagnosed with non small cell lung cancer (NSCLC) in many parts of the world, among which squamous cell carcinoma (SCC) and adenocarcinoma (ADC) are the two major histological subtypes [1]. Therefore, new discoveries of biomarkers for determining the risks of occurrence, progression and metastasis in NSCLC should have high clinical significance. The flotillin/reggie protein surperfamily contains two members, flotillin-1/reggie-2 (Flot-1) and flotillin-2/reggie-1 (Flot-2), both are highly conserved and both Flot-1 and Flot-2 proteins are ubiquitously expressed along with membrane rafts [2,3]. Furthermore, Flotillins have been suggested to be involved in membrane receptor signaling, signaling pathways associated with cell growth and malignant transformation, phagocytosis and endocytosis, cell matrix adhesion, regulation of actin cytoskeleton and formation of filopodia [4,5]. Expression of Flot-2 is interdependent and Flot-1 expression is severely reduced upon down regulation of Flot-2 [6]. Increased expression of Flotillins has been detected in several types of human cancer and linked with poor survival. Over-expression of Flot-2 is associated with human melanoma progression and lymph node metastasis [7,8]. Higher expression of Flot-2 also be detected in metastatic NPC cells [9]. Consistently, increased Flot-2 expression was also reported in NPC patients with lymph node metastasis compared with patients without lymph node metastasis [10]. Also, increased expression of Flot-2 is associated with poor outcomes in patients with several solid tumors, such as breast cancer, gastric cancer and cervical carcinoma, and Flot-2 could be used as a prognostic biomarker for these tumors progression [11–13].

The EGFR is a member of the receptor tyrosine kinase family involved in the regulation of cellular proliferation and differentiation which is directly collated with the mRNA transcript level of EGFR in NSCLC patients [14]. EGFR and its associated signaling pathway have emerged as a leading target for NSCLC therapy [15,16]. Interaction has been found between Flotillins and EGFR in cancer cells and Flot-2 becomes Tyr- phosphorylated by Src kinase based on EGFR activation [4]. Additionally, EGF stimulation results in uptake of Flotillins from the plasma membrane into late endosomes [4]. It not only mediates the clustering and activation of EGFR at the plasma membrane, but also functions as a MAP kinase scaffolding protein that regulates MAP kinase signaling during later stages of the pathway [17]. The expression of Flot-2 protein is up-regulated in the majority of NSCLC samples compared to the normal tissue [18,19]. However, dual expression of Flot-2 and EGFR and its relationship with development and progression or clinicopathologic/prognostic implication in large collection of NSCLC samples has not been reported. In this study, we detected Flot-2 and EGFR proteins by immunohistochemistry (IHC) in 352 cases of NSCLC and 59 cases of non-cancerous lung control tissues, and investigated the correlations between the expression of Flot-2 and EGFR proteins and clinicopathological features and prognostic implications in NSCLC.

## Materials and Methods

### Ethics Statement

Samples were obtained with informed consent and all protocols were approved by The Second Xiangya Hospital of Central South University Ethics Review Board (Scientific and Research Ethics Committee, no. s02/2000). Written informed consent was obtained from all patients, also the written informed consent was obtained from the next of kin, caretakers, or guardians on the behalf of the minors/children participants involved in your study.

### Tissue microarrays (TMA) and clinical data

NSCLC patients were submitted to surgical treatment at the Department of Thoracic Surgery at the Second Xiangya Hospital of Central South University (Changsha, China) from 2003 to 2013. All tumor samples and non-cancerous lung tissues were obtained from Department of Pathology, the Second Xiangya Hospital of Central South University. These patients had been submitted to routine staging and definitive surgical resection of the lung and systematic mediastinal lymph node dissection. All patients had a confirmed histological diagnosis of NSCLC according to WHO histological classification of the lung cancer. The staging classification of the current analysis was carried out based on the criteria of the 7th edition of the AJCC/UICC TNM staging system of lung cancer (2009). No patients had been previously treated with chemotherapy and radiotherapy at the time of original operation. Complete clinical record and follow-up data were available for all patients. Written informed consent was obtained from these patients, and this study was approved by the Ethics Review Committee of the Second Xiangya Hospital of Central South University.

### Immunohistochemistry and scores

The IHC staining for samples on the TMAs was carried out using ready-to-use Envision TM^+^ Dual Link System-HRP methods (Dako; Carpintrria, CA). The staining condition for each antibody was adjusted according to our laboratory experience. Briefly, each TMA section was deparaffinized and rehydrated, and high-temperature antigen retrieval was achieved by heating the samples in 0.01M citrate buffer in a domestic microwave oven at full power (750 Watts) for 30 minutes, then the samples were immersed into methanol containing 0.3% H_2_O_2_ to inactivate endogenous peroxidase at 37°C for 30 minutes. To eliminate nonspecific staining, the slides were incubated with appropriate preimmune serum for 30 minutes at room temperature. After incubation with a 1:1000 dilution of primary antibody to Flot-2 protein (Rabbit polyclonal antibody, Catalog: #S0051, Epitomics, Inc.) and with a 1:200 dilution of the primary antibody (Novocastra NCL-EGFR-384 clone, Leica Biosystems Newcastle, Newcastle Upon Tyne, UK) at 4°C overnight, slides were rinsed with phosphate-buffered saline (PBS) and incubated with a labeled polymer-HRP was added according to the manufacturer’s instructions and incubated 30 minutes. Color reaction was developed by using 3, 3’-diaminobenzidine tetrachloride (DAB) chromogen solution. All slides were counterstained with hematoxylin. Positive control slides were included in every experiment in addition to the internal positive controls. The specificity of the antibody was determined with matched IgG isotype antibody as a negative control.

Immunohistochemical staining of TMA sections were scored independently by QW and SF blinded to the clinicopathological data, at 200× magnification light microscopy. The evaluation was based on the staining intensity and extent of staining. Staining intensity for Flot-2 and EGFR was scored as 0 (negative), 1 (weak), 2 (moderate), and 3 (strong). Staining extent was scored as 0 (0%), 1 (1–25%), 2 (26–50%), 3 (51–75%), and 4 (76–100%), depending on the percentage of positive-stained cells. The sum of the staining intensity and the staining extent scores ranged from 0 to 7, and cut-off levels for Flot-2 and EGFR were chosen on the basis of a measure of heterogeneity using the log-rank test with respect to overall survival (OS). An optimal cut-off level was identified as follows: a staining index score of 0–2 was used to define tumors with negative expression and 3–7 indicated positive expression of these two proteins. Agreement between the two evaluators was 95%, and all scoring discrepancies were resolved through discussion between the two evaluators.

### Statistical analysis

The chi-square test and the Spearman's rank correlation coefficient were used to evaluate the relationship between the Flot-2 and EGFR expression in tissue sections harvested from NSCLC patients. Kaplan-Meier analysis was performed for overall survival curves and statistical significance was assessed using the log-rank test. Cox proportional hazard regression model was used to estimate the independent prognostic factor Flot-2 and EGFR proteins. Two-sided statistical analysis was used and the data were considered to be statistically significant when *P*<0.05.

## Results

### Flot-2 and EGFR proteins significantly increased in tissues of lung SCC and ADC

**Fig 1:**
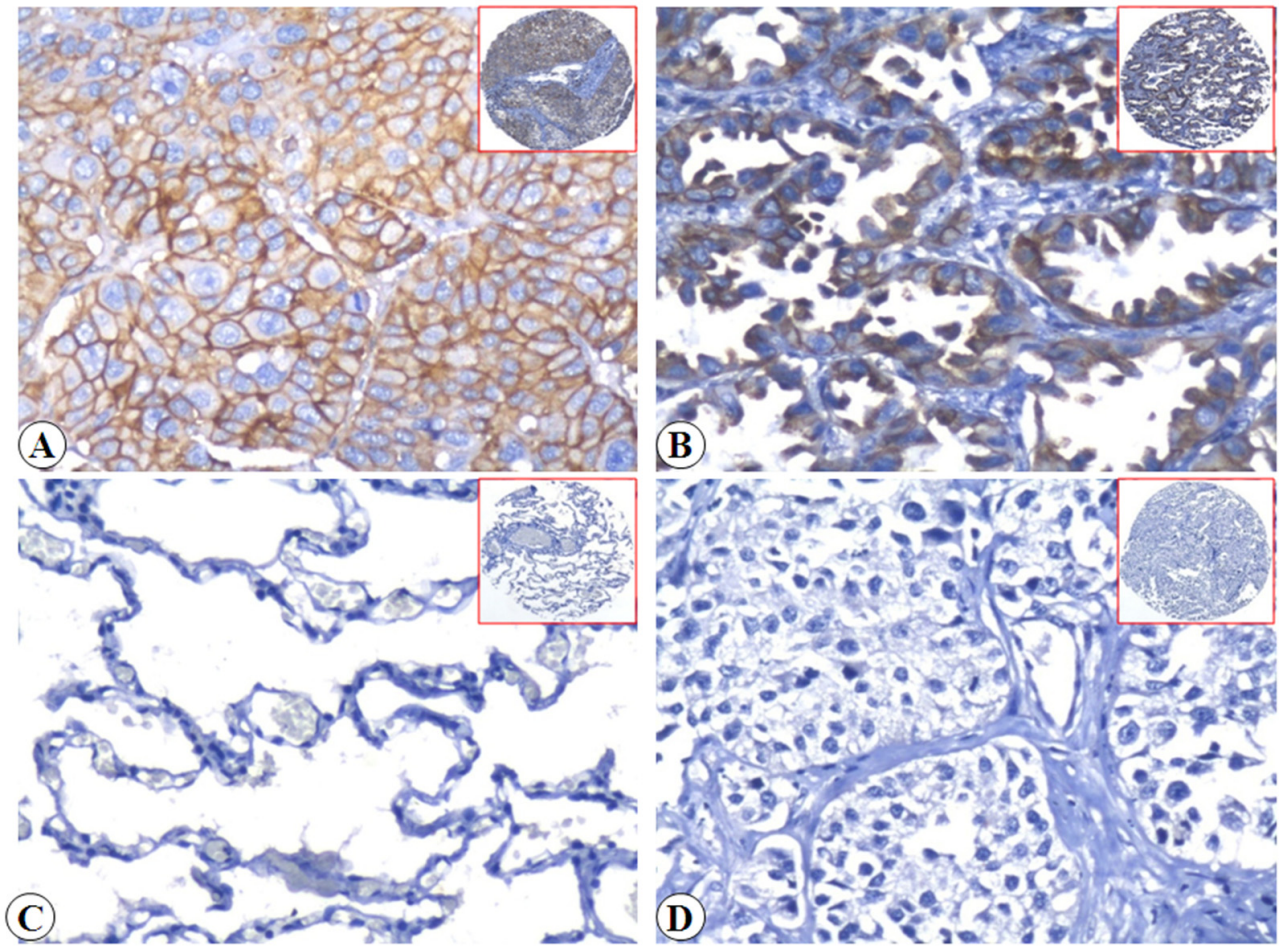
Expression of Flot-2 protein in lung SCC cells, lung ADC cells and control of non-cancerous lung tissues were detected by IHC using specific antibody as described in the section of materials and methods. Strong positive staining of Flot-2 protein was found on cell membranes of lung SCC and lung ADC cells (Fig 1A and 1B, 20×, IHC, DAB staining). Negative staining of Flot-2 was showed in non-cancerous lung tissue (Fig 1C, 20×, IHC, DAB staining). Negative control showed no Flot-2 staining in lung ADC cells (Fig 1D, 20×, IHC, DAB staining).

**Fig 2:**
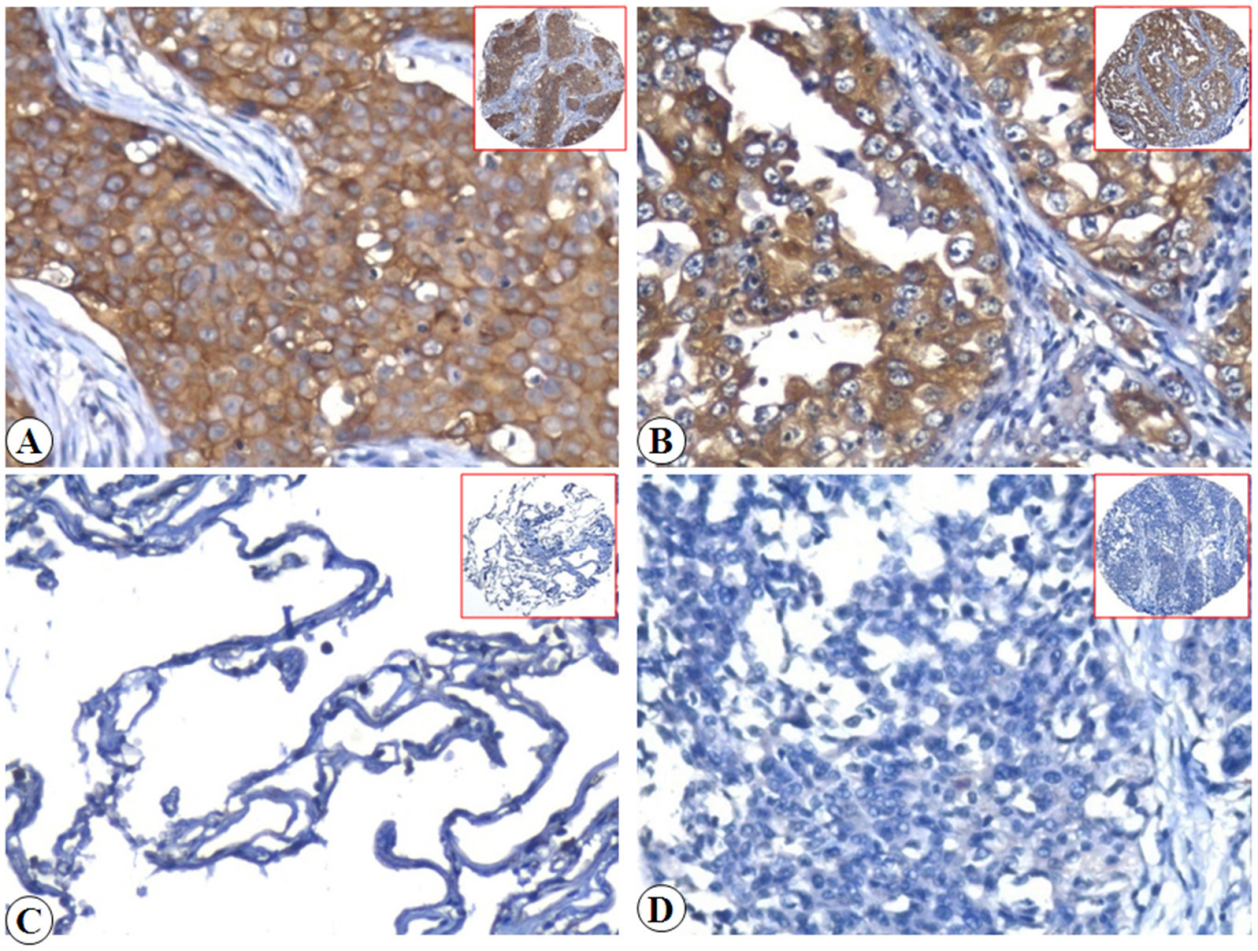
Expression of EGFR protein in lung SCC cells, lung ADC cells and the control of non-cancerous lung tissue were detected by IHC using specific antibody as described in the section of materials and methods. Strong positive staining of EGFR protein was found in cell membranes and cytoplasm of lung SCC and lung ADC cells (Fig 2A and 2B, 20×, IHC, DAB staining). Negative staining of EGFR was showed in non-cancerous lung tissue (Fig 2C, 20×, IHC, DAB staining). Negative control showed no EGFR staining in the lung SCC cells (Fig 2D, 20×, IHC, DAB staining).

**Fig 3:**
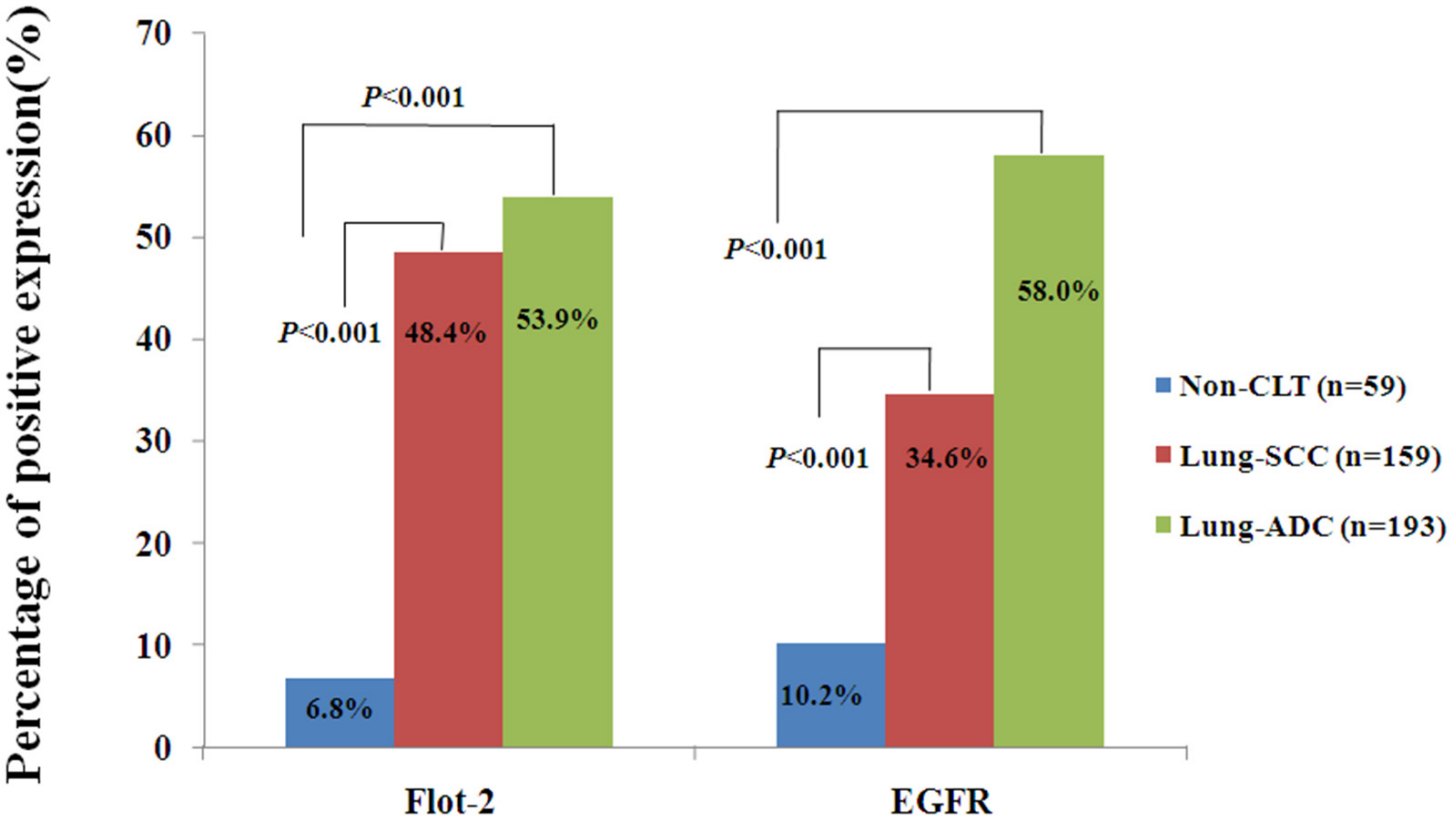
Expression of Flot-2 and EGFR proteins in lung SCC and lung ADC compared to the control of non-cancerous lung tissues. Results showed that there were significant differences between the groups which were statistically evaluated by chi-square test.

To explore the correlation between Flot-2 and EGFR signaling in malignant tissues, we first determined the expression and cellular localization of Flot-2 and EGFR in lung SCC, ADC and the non-cancerous lung control tissues by IHC. Strong positive expression of Flot-2 protein (Fig 1A and 1B) was identified on cell membranes of lung SCC and ADC tissues and no Flot-2 expressing cells were detected in non-cancerous lung control tissue (Fig 1C). Tissue sections using matched IgG isotype antibody as negative control also showed no positive staining of Flot-2 in the lung ADC cells (Fig 1D). Strong positive expression of EGFR protein (Fig 2A and 2B) was identified on membranes and cytoplasm of the lung SCC and ADC cells and no staining was found in the non-cancerous lung control tissue (Fig 2C). Negative control had shown no EGFR staining in the lung SCC cells (Fig 2D). Next, we enumerate the expression of Flot-2 and EGFR in lung SCC, ADC and non-cancerous control lung tissues. Expression of both Flot-2 and EGFR were significantly higher in tissues harvested from patients with lung SCC and ADC compared with the non-cancerous lung control tissues (*P*<0.001) (Fig 3).

To investigate the clinical outcome due to the over-expression of Flot-2 and EGFR in cancer tissues, we next investigated clinicopathological features of NSCLC including age, gender, clinical stages, lymph node metastasis (LNM) status, and pathological differentiation in univariate chi-square test. Date shown in Tables 1 and 2 indicate that a strong positive correlation exists between high expression of Flot-2 and the clinical stages of NSCLC. Importantly, patients with advanced clinical stages had significantly higher expression of Flot-2 than patients with early stages (*P* = 0.003). Similar trend was observed in case of EGFR expression (*P* = 0.03). However, no differences were observed between the expression of Flot-2/EGFR and other clinicopathological features such as age, gender, LNM status and pathological differentiation of NSCLC patients.

**Table 1:** 352 cases of non small cell lung cancer (NSCLC) patient’s characteristics.

Patients characteristics	No. of patients (%)
**Age(years)**	
≤50	100(28.4)
>50	252(71.6)
**Gender**	
Male	267(75.9)
Female	85(24.1)
**Clinical stages**	
I	76(21.6)
II	72(20.4)
III	170(48.3)
IV	34(9.7)
**Lymph node status**	
N0	141(40.1)
N1/N2/N3	211(59.9)
**Histological type**	
SCC	159(45.2)
ADC	193(54.8)
**Histological grades**	
Well differentiation	6(1.7)
Moderate differentiation	145(41.2)
Poor differentiation	201(57.1)

**Table 2:** Analysis of the association between expression of Flot-2 and EGFR proteins and clinicopathological features of NSCLC (n = 352).

Clinicopathological features (n)	Flot-2			EGFR			Flot-2/ EGFR^#^		
	P (%)	N (%)	P-value	P (%)	N (%)	P-value	P^+^ (%)	N^-^ (%)	P-value
**Age(years)**									
≤50 (n = 100)	47(47.0)	53(53.0)		47(47.0)	53(53.0)		61 (61.0)	39(39.0)	
>50 (n = 252)	134(53.2)	118(46.8)	0.344	120(47.6)	132(52.4)	1.000	172(68.3)	80(31.7)	0.212
**Gender**									
Male(n = 267)	140(52.4)	127(47.6)		123(46.1)	144(53.9)		176(65.9)	91(34.1)	
Female(n = 85)	41(48.2)	44(51.8)	0.535	44(51.8)	41(48.2)	0.384	57(67.1)	28(32.9)	0.896
**Clinical stages**									
Stage _I-II_ (n = 148)	62(41.9)	86(58.1)		69(46.6)	79(53.4)		88(59.5)	60(40.5)	
Stage _III-IV_ (n = 204)	119(58.3)	85(41.7)	0.003*	98(48.0)	106(52.0)	0.829	145(71.1)	59(28.9)	0.03*
**LNM status**									
LNM (n = 211)	111(52.6)	100(47.4)		104(49.3)	107(50.7)		142(67.3)	69(32.7)	
No LNM (n = 141)	70(49.6)	71(50.4)	0.589	63(44.7)	78(55.3)	0.446	91(64.5)	50(35.5)	0.646
**Histological type**									
SCC(n = 159)	77(48.4)	82(51.6)		55(34.6)	104(65.4)		95(59.7)	64(40.3)	
ADC(n = 193)	104(53.9)	89(46.1)	0.336	112(58.0)	81(42.0)	0.000*	138(71.5)	55(28.5)	0.024*
**Histological grades**									
Well (n = 6)	3(50.0)	3(50.0)		4(66.7)	2(33.3)		5(83.3)	1(16.7)	
Moderate(n = 145)	69(47.6)	76(52.4)		76(50.3)	75(49.7)		96(66.2)	49(33.8)	
Poor (n = 201)	109(54.2)	92(45.8)	0.474	91(45.3)	110(54.7)	0.460	132(65.7)	69(34.3)	0.666

*Chi-square test, statistically significant difference (*P*<0.05)

Abbreviations: LNM, lymph node metastasis; SCC, squamous cell carcinoma; P, Positive; N, Negative;

^#^P^+^, positive expression with either of Flot-2 and EGFR proteins; P^-^, common negative staining of the two proteins.

### The pairwise association between expression of Flot-2 and EGFR proteins in 352 cases of NSCLC

We next analyzed the pair-wise association between expression of Flot-2 and EGFR in NSCLC patients (n = 352). Patients (n = 115) with co-expression of Flot-2 and EGFR proteins, 119 patients with common negative staining of two proteins and 118 patients with positive expression of either Flot-2 or EGFR. The increased expression of Flot-2 was significantly associated with the increased expression of EGFR (r = 0.332, *P*<0.001 spearman rank correlation test). These data suggest that Flot-2 and EGFR might play an important role in promoting the development and progression of NSCLC.

### Impact of expression of Flot-2 and EGFR proteins on the prognosis of patients with NSCLC

**Fig 4:**
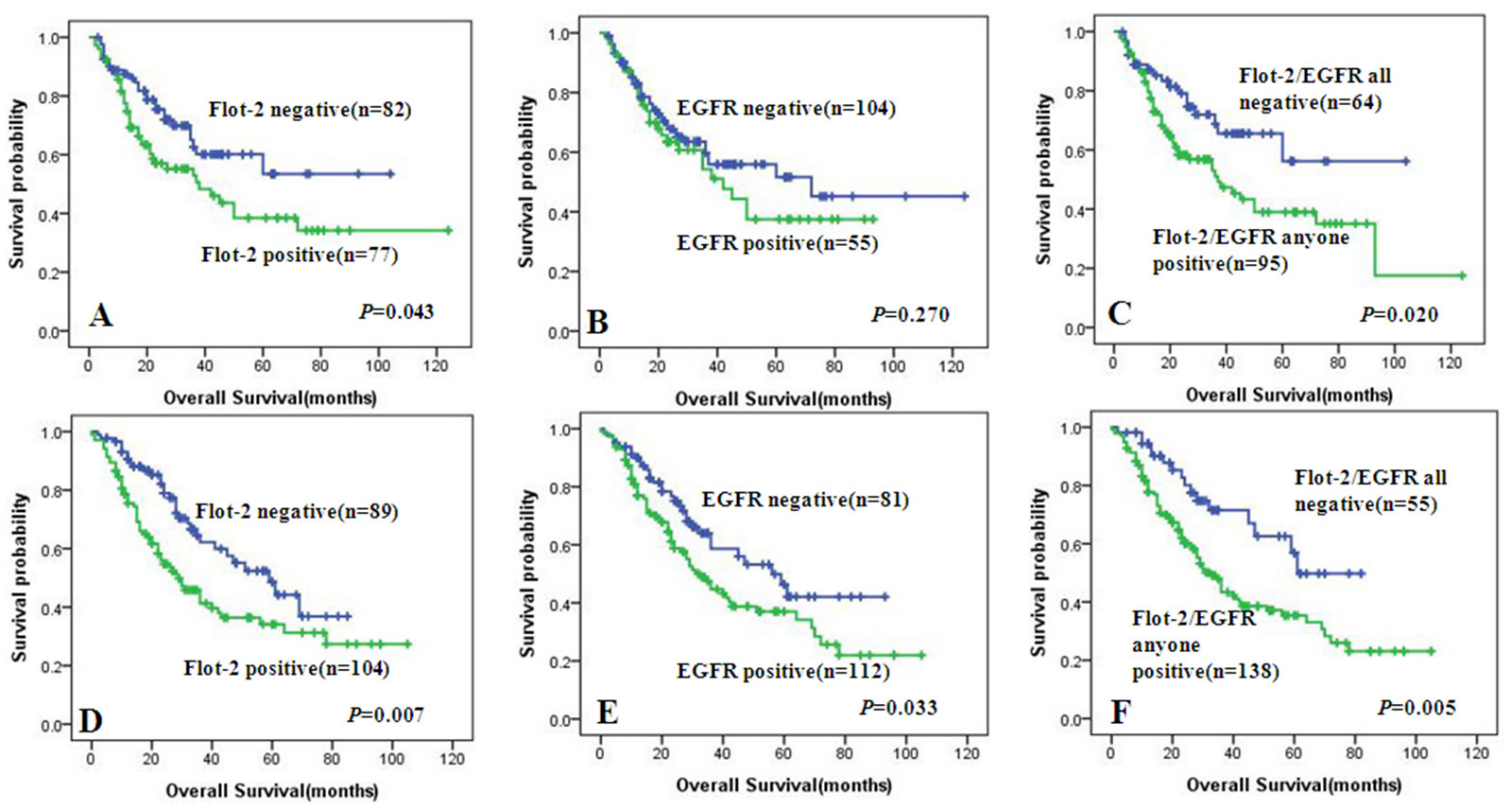
Kaplan-Meier cures for overall survival of lung SCC and ADC patients with expression of Flot-2 and EGFR. Kaplan-Meier analysis was used to plot the overall survival curves of 159 cases of lung SCC and 193 cases of lung ADC patients with differential expression of Flot-2, EGFR, and combined expression of either of these two proteins, which statistical significance was assessed by log-rank test. (A) Lung SCC patients with positive expression of Flot-2 protein showed worse overall survival rates compared to patients with negative Flot-2 (P = 0.043, two sided). (B) Positive expression of EGFR had no significantly correlation with overall survival rates of lung SCC patients (P> 0.05, two sided). (C) Kaplan-Meier curves showed lung SCC patients with positive expression with either of Flot-2 and EGFR proteins had worse overall survival rates than these with all negative staining of two proteins above (P = 0.02, two sided). (D) Lung ADC patients with positive expression of Flot-2 had worse overall survival rates than that with negative one (P = 0.007, two sided, respectively). (E) Lung ADC patients with positive expression of EGFR had worse overall survival rates than that with negative one (P = 0.033, two sided, respectively). (F) Lung ADC patients with positive expression with either of Flot-2 and EGFR proteins showed worse overall survival rates compared with all negative staining of two proteins above (P = 0.005, two sided).

In univariate survival analysis of lung SCC and ADC patients, Kaplan-Meier survival curve analysis with log-rank significance test was performed. The Kaplan-Meier survival plots (Fig 4A, 4B and 4C) for lung SCC patients with differential expression of Flot-2 (Fig 4A), EGFR (Fig 4B), and combined expression of either of these two proteins (Fig 4C) were presented. The Kaplan-Meier survival plots for lung ADC patients with differential expression of Flot-2 (Fig 4D), EGFR (Fig 4E), and combined expression of either of these two proteins (Fig 4F) were presented. The overall survival rates for lung SCC and ADC patients with negative expression of Flot-2 protein were significantly higher than these with positive Flot-2 expression (*P* = 0.043, Fig 4A; *P* = 0.007, Fig 4D), as well as the overall survival rates for lung ADC patients with negative expression of EGFR were better than these with positive EGFR expression (*P* = 0.033, Fig 4E). In addition, those lung SCC and ADC patients with positive expression with either of Flot-2 and EGFR proteins had a lower overall survival rates than patients with all negative staining of two proteins above (*P* = 0.02, Fig 4C; *P* = 0.005, Fig 4F). However, no significant association between expression of EGFR and overall survival rates were noticed in lung SCC patients (*P* >0.05).

Besides univariate analysis, multivariate Cox proportional hazard regression analysis was also carried out to further investigate whether the expressions of Flot-2 and EGFR proteins are the independent prognostic factors for NSCLC, and these results were revealed in Table 3. During the multivariate analysis of the expression of Flot-2 and EGFR proteins in 159 lung SCC cases and 193 lung ADC cases, which included clinical stages, lymph node metastasis status, histological type, treatment strategy, age and gender, we have found that positive expression of Flot-2 protein may serve as an independent poor prognostic factor for lung ADC (*P* = 0.035), as well as clinical stages (*P* = 0.02). For lung SCC, LNM status and histological grades are identified as independent poor prognostic factors (*P* = 0.034, *P* = 0.019, respectively). Again, no clinical effect based on age, gender, treatment strategy and expression of EGFR was detected in lung SCC and ADC.

**Table 3:** Summary of multivariate analysis of Cox proportional regression for overall survival in 352 cases of NSCLC.

Histological type	Lung SCC				Lung ADC			
Parameter			95.0%CI for Exp(B)				95.0%CI for Exp(B)	
	Sig.	Exp(B)	Lower	Upper	Sig.	Exp(B)	Lower	Upper
**Age**	0.999	0.997	0.567	1.754	0.679	0.910	0.582	1.424
**Gender**	0.131	0.331	0.079	1.392	0.805	1.056	0.688	1.620
**LNM status**	0.034*	1.872	1.047	3.348	0.278	1.326	0.796	2.208
**Clinical stages**	0.069	1.878	0.953	3.704	0.020*	1.803	1.095	2.968
**Histological grades**	0.019*	1.954	1.118	3.415	0.272	1.274	0.827	1.961
**Treatment strategy**	0.518	1.232	0.654	2.322	0.549	1.176	0.692	1.999
**Flot-2 expression**	0.576	1.162	0.687	1.966	0.035*	1.657	1.036	2.650
**EGFR expression**	0.308	1.304	0.782	2.173	0.225	1.340	0.835	2.149

Abbreviations: LNM, lymph node metastasis; SCC, squamous cell carcinoma; ADC, adenocarcinoma; CI, confidence interval.

Note: multivariate analysis of Cox regression,

*P<0.05.

## Discussion

Increasing evidence has indicated that Flotillins involved in EGFR signaling pathway. Activated EGFR could change the size of Flotillins oligomers [20]. Additionally, Flotillins and EGFR proteins are coimmunoprecipitated, and Flotillins could regulate E-cadherin-mediated cell contact formation by affecting EGFR trafficking [17,21]. In our present study, we have reported that expressions of Flot-2 and EGFR were significantly increased in lung SCC and ADC compared with the non-cancerous lung control tissues. The increased expression of Flot-2 was significantly associated with the increased expression of EGFR in NSCLC when Spearman's rank correlation coefficient was carried out. Furthermore, NSCLC patients with advanced clinical stages had significant higher positive percentage of Flot-2 expression than those with earlier stages. Above all, our results are not only consistent with previously published data [18,19], but also bring people new field of vision. Firstly, over-expression of both Flot-2 and EGFR were found in NSCLC; Secondly, positive relationship between the expression of Flot-2 and EGFR in NSCLC absolutely existed; At last, Flot-2 and EGFR might play an important role in promoting the development and progression of NSCLC. Nonetheless, only this evidence is not enough to draw a strong conclusion; hence further investigation will be needed to clearly demonstrate the specific mechanism of Flot-2 and EGFR in NSCLC, and that’s what we’re about to study in the near future.

Although the tumor-node-metastasis (TNM) staging system is the best prognostic index for operable NSCLC [22], biomarkers do not only play overwhelmingly important role in establishing an accurate diagnosis, but also providing prognostic data for NSCLC [23]. Therefore, new discoveries of biomarkers for determining the risks of occurrence, progression and approaches for therapeutic treatment of NSCLC are of extreme importance for the development of therapeutic strategies to improve outcome and survival of NSCLC patients. Flot-2, as a target gene of p63 and p73, member of the p53 transcription factor family [24], has been proposed as a prognostic marker linked to poor prognosis in several human tumors, such as breast cancer, gastric cancer, cervical carcinoma and so on [11–13]. However, the significance of Flot-2 in lung cancer was still seldom reported. Previous literature showed that only two investigations about Flot-2 that have been conducted in NSCLC till today. But they have either no observation about the association between over-expression of Flot-2 and clinical outcome as well as poor survival or no description about research on the expression pattern of EGFR associated with Flot-2 in lung SCC and lung ADC, respectively [18,19]. In our current study, we showed that both lung SCC and ADC patients with increased expression of Flot-2 had worse overall survival rates compared with patients with negative Flot-2. Furthermore, multivariate analysis proved that the increased expression of Flot-2 was the independent factor for poor prognosis in lung ADC patients regardless of clinical stages, histological type, LNM status, histological grades, age and gender. Our results are not only in accordance with or similar with the previously published data, but also suggest that high expression of Flot-2 might participate in promoting cell survival and proliferation and associate with the poor prognosis of lung ADC patients. Therefore, increased expression of Flot-2 might be considered as a novel biomarker to predict poor prognosis for lung ADC patients.

As far as EGFR is concerned, many previous studies have suggested that expression of high levels of EGFR is associated with progress, metastasis and poor prognosis of cancer patients, but the prediction power of this single receptor for patients’ outcome is controversial. The reports about co-expression of EGFR and other proteins in NSCLC have a positive synergistic effect on patients’ outcome [25,26]. In our present study, positive expression of EGFR had significantly correlation with overall survival rates for lung ADC patients. Furthermore, lung SCC and ADC patients with positive expression of either of Flot-2 and EGFR proteins had a lower overall survival rates than patients with all negative staining of these two proteins. Therefore, our results suggest an important interaction between Flot-2 and EGFR intracellular pathway in the progression of NSCLC and high expression of Flot-2 and EGFR proteins might have a positive synergistic effect on patients’ outcome.

In conclusion, our finds demonstrated a positive correlation between Flot-2 and EGFR levels in NSCLC tissues. Expression level of Flot-2 protein has correlated with poor prognosis both in lung SCC and ADC patients suggesting that Flot-2, associated with increased EGFR may serve as biomarker for indicating poor prognosis and potential therapeutic target for NSCLC.
